# Prevalence of Obstructive Sleep Apnea and Adherence to CPAP for TAXI Drivers

**DOI:** 10.3390/clockssleep8010004

**Published:** 2026-01-07

**Authors:** Yik Hin Chan, Anastasya Maria Kosasih, Venetia Kok, Yi-Hui Ou, Yun Jing Crystal Chng, Joshua J. Gooley, Chi-Hang Lee

**Affiliations:** 1 Department of Cardiology, National University Heart Centre, 1 E Kent Ridge Road, NUHS Tower Block Level 9, Singapore 119228, Singapore; 2 Neuroscience and Behavioural Disorders Programme, Duke-NUS Medical School, Singapore 169857, Singapore; 3 Department of Sleep Medicine, Alexandra Hospital, Singapore 159964, Singapore

**Keywords:** obstructive sleep apnea, blood pressure, psychomotor vigilance test, continuous positive airway pressure

## Abstract

We investigated the effects of Continuous Positive Airway Pressure (CPAP) on blood pressure (BP) and vigilance in taxi drivers with obstructive sleep apnea (OSA). This pilot study recruited taxi drivers aged ≥60 years to undergo polysomnography. Those diagnosed with OSA underwent 6 months of CPAP therapy. Baseline and follow-up assessments included 24 h ambulatory blood pressure monitoring (ABPM) and the psychomotor vigilance test (PVT). Among the 32 participants, 22 (68.8%) were diagnosed with OSA (median age 63.0 [62.0–65.0] years; 21 males). The average CPAP adherence was 3.1 ± 2.3 h per night, with 23.5% using CPAP for more than 4 h per night. There were no significant changes in 24 h mean systolic ABPM (125.9 [116.8–134.9] mmHg to 126.0 [118.3–133.7] mmHg; *p* = 0.93) or reaction times measured by PVT (2.0 [0.0–3.0] lapses to 2.0 [1.0–3.0] lapses; *p* = 0.82) after CPAP therapy. A high prevalence of OSA was observed among taxi drivers. CPAP adherence was suboptimal and did not result in significant improvements in BP or vigilance.

## 1. Introduction

Drowsy driving has become increasingly recognized as a major contributor to road traffic accidents, accounting for 1.6% of fatalities in vehicle crashes [1]. One of the key factors in sleep-deprived driving is obstructive sleep apnea (OSA), a condition that significantly disrupts sleep and leads to excessive daytime sleepiness [2]. People who commonly work long hours with irregular schedules, such as taxi drivers, are particularly vulnerable to OSA. Their sedentary lifestyle, combined with reduced physical activity, contributes to increased rates of obesity, further elevating OSA risk [3].

Globally, road traffic accidents cause the death of 1.19 million people each year and one of the causes is drowsy driving, which contributes to around 21% of fatal crashes [4]. The National Highway Traffic Safety Administration (NHTSA) reported that about 91,000 crashes in 2017 in the U.S. were linked to drowsiness. However, the real impact may be underreported, making the global toll likely higher [5]. Drivers with undiagnosed OSA have 2.5 times higher risk of experiencing drowsiness while driving, significantly raising the probability of serious accidents that endanger the safety of drivers, passengers, and other road users [6]. This situation is caused by frequent disruptions in breathing during sleep, resulting in sleep fragmentation and preventing restorative sleep. Collapse of the airway leads to momentary awakening, preventing individuals from reaching deeper sleep stages and causing vigilance decrements [7]. Today, cognitive impairment during obstructive sleep apnea is considered not only a consequence of direct disturbances in total sleep duration and daytime sleepiness, but also a consequence of global neurophysiological processes [8]. Moreover, OSA causes intermittent hypoxia, which activates the sympathetic nervous system, leading to an increase in blood pressure (BP) during sleep [9]. In patients with OSA, continuous positive airway pressure (CPAP), the first-line treatment, may help regulate BP, improve vigilance, and enhance sleep quality [10].

This study aims to assess the effects of CPAP therapy in taxi drivers diagnosed with OSA in terms of BP, vigilance levels, and sleep quality. This may improve their employability without compromising road safety.

## 2. Results

Among 32 participants who underwent overnight polysomnography, 68.8% (22 out of 32) had OSA, including 90.9% (20 out of 22) who were diagnosed with moderate-to-severe OSA, and 9.1% (2 out of 22) who were identified with mild OSA. The median age was 63.0 [62.0–65.0] years, with 95.4% (21 out of 22) males, and the median BMI was 27.7 [25.6–31.6] kg/m^2^. The median AHI was 35.1 [20.0–47.9] events per hour. Hypertension was the most prevalent cardiovascular risk factor, observed in 63.6% of patients, followed by hyperlipidemia in 59.0%, and type 2 diabetes mellitus in 40.9%. Daytime sleepiness severity, assessed by the ESS, revealed that 13.6% of participants were classified as mildly sleepy, 4.5% as moderately sleepy, and 9.0% as severely sleepy. Further details of these 22 participant could be found in (Supplementary Table S1). 

At the 6-month follow-up, 18 participants completed the study. Adherence to CPAP therapy defined as using the device for ≥4 h per day on ≥70% of days, was 23.5% (4 out of 17) with a mean usage of 3.1 ± 2.3 h per night. One participant’s data was unavailable due to a CPAP machine error.

The change in systolic 24 h ABPM from baseline (125.9 [116.8–134.9] mmHg) to the 6-month follow-up (126.0 [118.3–133.7] mmHg) was not statistically significant (*p* = 0.93). Likewise, there were no differences in systolic office BP (135.6 [127.7–143.0] mmHg to 132.1 [122.6–141.5] mmHg, *p* = 0.39), awake systolic BP (127.9 [119.7–136.1] mmHg to 128.2 [120.3–136.1] mmHg, *p* = 0.83) or asleep systolic BP (121.3 [110.6–132.0] mmHg to 120.8 [112.9–128.7] mmHg, *p* = 0.82) from baseline to the 6-month follow-up (Table 1). Moreover, no significant association was observed between CPAP adherence, CPAP usage times, and blood pressure outcomes in 6 months.

**Figure 1:**
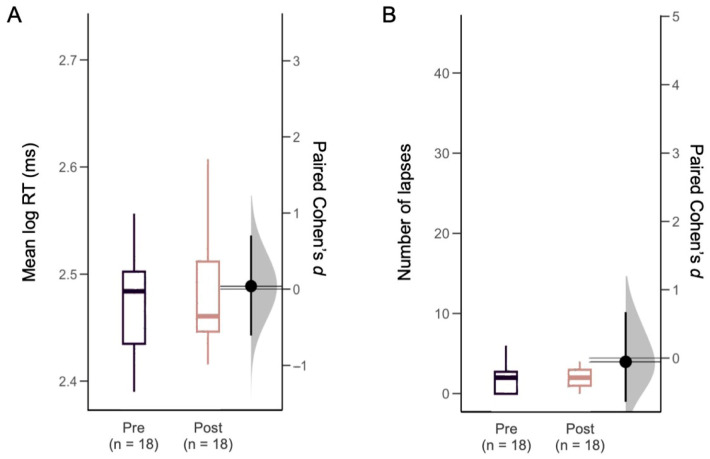
Effect size of pre- and post-CPAP treatment in (**A**) PVT mean log-transformed reaction time (log RT) and (**B**) PVT lapses (RT > 500 ms).

Visual attention performance did not differ from baseline to 6 months after CPAP treatment, as measured by PVT RT (294.7 [260.0–316.0] ms to 283.7 [269.0–323.0] ms, *p* = 0.74) and lapses (2.0 [0.0–3.0] lapses to 2.0 [1.0–3.0] lapses, *p* = 0.82). Moreover, no significant effect was observed in the mean log-transformed RT (and the number of lapses (RT > 500 ms) after CPAP treatment (Figure 1). In the LMMs that adjusted for covariates (age, BMI, chronic disease, sleep apnea severity, nocturnal sleep duration, and CPAP compliance), there was no change in PVT log RT (β = 0.00 log ms, 95% CI = −0.01 to 0.01) or lapses (β = −0.50 lapses, 95% CI = −2.31 to 1.31) after CPAP treatment (Supplementary Tables S2 and S3). Subjective ratings also did not change for alertness on the VAS (60.5 [41.0–79.5] to 65.1 [43.0–77.8], *p* = 0.96), or for sleepiness on the KSS (4 [3–5] to 3 [3–4], *p* = 0.30) after CPAP treatment (Table 1). Linear Mixed Model between PVT lapses and age, body mass index, chronic disease status, sleep apnea severity, average sleep time, CPAP compliance, and condition are shown in Supplementary Tables S4.

## 3. Discussion

This study assessed the effects of a 6-month CPAP intervention on BP and vigilance in elderly taxi drivers in Singapore with OSA. This group is particularly relevant because untreated OSA can severely impact driving safety. However, research on CPAP effectiveness in this demographic is limited due to small sample sizes and low adherence to CPAP therapy.

Our findings indicated no change in 24 h systolic ABPM or systolic office BP after CPAP use. By comparison, prior studies have shown benefits of CPAP in BP management for OSA patients [3]. The lack of statistical significance in our study could be attributed to the small sample size and poor CPAP adherence [11,12], averaging only 3.1 h per night, likely influenced by participants’ irregular schedules.

In our study, attentional performance on the PVT and self-rated alertness did not improve after CPAP treatment. Previous studies have shown that high use of CPAP lowers daytime sleepiness [13], which has important implications for taxi drivers with OSA [14]. However, previous studies have also reported marginal improvement in steering performance on driving simulators after 3 months of CPAP, with no change in RT. The absence of significant differences might be due to persistent neurocognitive effects from hypoxia, genetic predispositions, or insufficient CPAP adherence to yield full benefits [15]. Several epidemiological studies have indicated a decrease in motor vehicle accident rates among OSA patients undergoing CPAP treatment [16]. Additionally, longer CPAP usage duration has been linked to a further reduction in accidents. However, it remains unclear whether other factors, such as behavioral changes or additional treatments, also contribute to the observed decrease in accident rates [17].

The majority of the taxi drivers in this study (90.9%) had moderate-to-severe OSA. OSA severity can lead to increased sleepiness, impaired attention, and deficits in information processing, potentially resulting in risky driving behaviors such as speeding and running red lights. Sleepiness may also cause memory lapses and fatigue, negatively affecting mood and leading to aggressive driving [18]. In our pilot study of taxi drivers, however, subjective and objective measures of vigilance did not change after 6 months of CPAP use.

Although the proportion of sleep time during which CPAP was worn may have been reasonable, the overall duration of sleep was short, thereby reducing the total therapeutic exposure to CPAP. This pattern is consistent with the occupational demands of taxi drivers, who frequently work long, irregular shifts, resulting in delayed bedtimes, early awakenings, and fragmented sleep. Potential strategies to improve sleep time and treatment adherence include work-schedule adjustments to allow more consistent rest periods, sleep hygiene counseling aimed at optimizing the limited sleep opportunities available, educational programs for drivers and employers regarding the cardiovascular and safety risks associated with untreated OSA and inadequate sleep, and consideration of alternative treatment modalities (e.g., mandibular advancement devices) for individuals who unable to tolerate CPAP [19].

This study focused on taxi drivers aged ≥60 years, a population that remains highly active in contemporary society and represents a group at elevated risk for OSA–related driving impairment. Given the high prevalence of OSA in older adults and its relationship with acute coronary event [20,21], the findings have potential relevance to road safety and occupational health. However, the small sample size limits statistical power and generalizability. In addition, suboptimal adherence to CPAP therapy among taxi drivers may have attenuated the observed treatment effects.

## 4. Methods and Materials

The study recruited 32 taxi drivers aged ≥60 years from a taxi company in Singapore to undergo polysomnography. Individuals with OSA who were already receiving treatment were excluded. Baseline measurements were collected, including height, weight, office blood pressure (BP), hip and waist circumference, and current medications.

Participants completed the Epworth Sleepiness Scale (ESS) to assess subjective daytime sleepiness before undergoing an overnight level 1 polysomnography sleep study. Polysomnographic recordings were scored manually according to the American Academy of Sleep Medicine (AASM) scoring criteria version 2.6 by specialist sleep technologist. ESS scores were categorized into four groups: non-sleepy (0–10), mildly sleepy (11–14), moderately sleepy (15–17), and severely sleepy (18–24).

OSA was diagnosed based on an Apnea-Hypopnea Index (AHI) of ≥15 events per hour, indicating moderate to severe OSA, or an AHI of 5–15 events per hour combined with an ESS score ≥ 10, indicating mild OSA. Participants without OSA (AHI ≤ 5) and with ESS scores < 10 were considered to have completed the study.

Those diagnosed with OSA continued in the study (n = 22), beginning with 24 h ambulatory blood pressure monitoring (ABPM). During this period, participants kept an activity diary, recording their hourly activities to correlate with BP fluctuations.

After the PSG and ABPM, participants visited the clinic and completed a 10 min Psychomotor Vigilance Test (PVT) to measure visual attention performance. PVT outcome measures included the mean of log-transformed reaction times (log RT) and the number of instances where participants responded slower than 500 milliseconds (lapses). Self-rated mood was assessed using Visual Analog Scales (VAS; scale 0–100) where participants were presented with the word pairs sad-happy, alert-sleepy, and calm-excited. Sleepiness was also evaluated using the Karolinska Sleepiness Scale (KSS). Participants were provided with CPAP treatment, and after 6 months, they returned for follow-up evaluations, including ABPM and PVT, to test for improvements following the treatment period. The study was approved by the institutional review board (Domain Specific Review Board—C, 2022-00062, National Healthcare Group). All participants provided informed consent.

The sample characteristics were summarized using mean ± SD, median and interquartile range (Q1–Q3), and frequency (%). Changes from baseline to the 6-month outcome were assessed with a paired *t*-test or Wilcoxon signed-rank test, and the effect size was measured by Cohen’s d for paired samples. Linear mixed models (LMM) were used to test for longitudinal changes in PVT performance, adjusting for covariates including CPAP adherence. All statistical analyses were performed with Stata BE version 18.0 (Stata Corp LLC, College Station, TX, USA).

## 5. Conclusions

There was a high prevalence (68.8%) of OSA among taxi drivers. CPAP treatment in elderly taxi drivers with OSA showed no significant improvements in BP or vigilance. The adherence level to CPAP may be a key factor in determining the effects on BP and vigilance. Additionally, the majority of participants exhibited only mild sleepiness, which could further explain the lack of observed improvements in vigilance. Further research is required with a larger sample size, improved CPAP adherence, and a focus on whether these improvements correlate with a sustained reduction in real-life accident risks.

## Figures and Tables

**Table 1:** PVT and BP Changes from Baseline to 6-Month Follow-Up.

	Baseline (n = 18)	6 Months (n = 18)	Difference (95% CI)	*p* Value
Office BP, mmHg *				
Mean BP	96.0 (90.7–101.2)	93.3 (86.6–100.0)	2.7 (−3.0 to 8.3)	0.33
Systolic BP	135.6 (127.7–143.0)	132.1 (122.6–141.5)	3.5 (−5.1 to 12.2)	0.39
Diastolic BP	76.1 (71.5–80.7)	73.9 (67.8–80.0)	2.22 (−2.7 to 7.2)	0.36
24 h, mmHg *				
Mean BP	95.5 (89.1–102.0)	95.2 (89.9–100.5)	0.3 (−2.0 to 2.7)	0.77
Systolic BP	125.9 (116.8–134.9)	126.0 (118.3–133.7)	−0.1 (−3.0 to 2.8)	0.93
Diastolic BP	82.2 (77.4–87.0)	79.8 (75.3–84.3)	2.4 (0.6 to 4.2)	0.01
Pulse pressure	45.5 (40.5–41.6)	46.2 (41.6–50.9)	0.7 (−3.1 to 1.6)	0.53
Awake, mmHg *				
Mean BP	97.4 (91.8–103.1)	97.0 (91.7–102.2)	0.4 (−1.4 to 2.4)	0.60
Systolic BP	127.9 (119.7–136.1)	128.2 (120.3–136.1)	−0.2 (−3.1 to 2.5)	0.83
Diastolic BP	82.2 (77.4–87.0)	81.3 (77.0–85.7)	0.8 (−1.6 to 2.8)	0.35
Pulse pressure	45.6 (40.4–50.8)	46.8 (41.7–51.9)	−1.1 (−3.7 to 1.4)	0.34
Asleep, mmHg *				
Mean BP	91.6 (83.2–99.9)	90.5 (84.3–96.7)	1.1 (−3.5 to 5.6)	0.63
Systolic BP	121.3 (110.6–132.0)	120.8 (112.9–128.7)	0.5 (−4.5 to 5.6)	0.82
Diastolic BP	76.6 (69.3–83.9)	76.2 (70.7–81.7)	0.4 (−4.5 to 5.4)	0.85
Pulse pressure	44.8 (40.1–49.4)	44.7 (40.6–48.7)	0.1 (−2.1 to 2.3)	0.93
**KSS**	4 (3–5)	3 (3–4)		0.30
**VAS**				
Sad (0)–Happy (100)	64.9 (54.6–73.7)	61.7 (52.0–80.0)		0.66
Alert (0)–Sleepy (100)	60.5 (41.0–79.5)	65.1 (43.0–77.8)		0.96
Calm (0)–Excited (100)	51.7 (46.1–64.8)	54.6 (47.2–69.1)		0.81
**PVT, lapses**	2.0 (0.0–3.0)	2.0 (1.0–3.0)		0.82
**PVT RT, ms**	294.7 (260.0–316.0)	283.7 (269.0–323.0)		0.75

* Values represent in mean (95% Confidence interval).

## Data Availability

The raw data supporting the conclusions of this article will be made available by the authors on request.

## Supplementary Materials

The following supporting information can be downloaded at: https://www.mdpi.com/article/10.3390/clockssleep8010004/s1.
