# Supplementary material for: Prevalence of Obstructive Sleep Apnea and Adherence to CPAP for TAXI Drivers
Source: Clocks Sleep. 2026 Jan 7;8(1):4. doi: 10.3390/clockssleep8010004 (PMC12821386; doi:10.3390/clockssleep8010004)
Supplement: Supplementary file 1 [file clockssleep-08-00004-s001.zip › clockssleep-3998876-supplementary.pdf]

SUPPLEMENTARY FILES

Supplementary Table S1. Baseline, clinical characteristic, ESS, and polysomnography

**Patient Characteristics (n=22)**	
Age, y	63.0 (62.0 – 65.0)
Male	21 (95.4%)
Height, cm	168.5 (164.0 – 173.0)
Weight, kg	80.3 (70.9 – 92.6)
BMI, kg/m^2	27.7 (25.6 – 31.6)
Neck circumference, cm	40.0 (38.0 – 42.0)
Waist circumference, cm	99.5 (93.5 – 112.0)
Hip circumference, cm	100.7 (97.7 – 108.0)
Waist/hip ratio	0.9 (0.9 - 1.0)
**Cardiovascular risk features**	
Hypertension	14 (63.6%)
Diabetes mellitus	9 (40.9%)
Previous stroke	2 (9.0%)
Coronary artery disease	1 (4.5%)
Chronic kidney disease	1 (4.5%)
Smoker	1 (4.5%)
Hyperlipidemia	13 (59.0%)
Previous PCI	1 (4.5%)
**Medications**	
Aspirin	3 (13.6%)
Beta-blocker	6 (27.2%)
ACE-i/ ARB	11 (50.0%)
Calcium-channel blocker	9 (40.9%)
Diuretic	4 (18.1%)
Statin	11 (50.0%)
**Daytime sleepiness severity (n=22)**	
Non-sleepy, ESS 0-10	16 (72.7%)
Mildly sleepy, ESS 11-14	3 (13.6%)
Moderately sleepy, ESS 15-17	1 (4.5%)
Severely sleepy, ESS 18-24	2 (9.0%)
**AHI 3%, events per hour (n=22)**	35.1 (20.0 - 47.9)
>/= 15 events per hour	20 (90.9%)
< 15 events per hour	2 (9.0%)
**ODI 3%, events per hour (n=22)**	33.4 (17.5 – 47.2)
>/= 15 events per hour	18 (81.8%)
< 15 events per hour	4 (18.1%)

Supplementary Table S2. LMM between PVT RT and age, BMI, chronic disease status, sleep apnea severity, average sleep time, CPAP compliance, and condition
Fixed effects

	**Estimate (95% CI)**	**SE**	***t*(df)**	***P***
**Intercept**	2.54 (2.48 to 2.59)	0.033	77.24(10.14)	**<0.001**
**Age at enrolment**				
<63 years old	-0.04 (-0.10 to 0.01)	0.036	-1.20(10.00)	0.257
63 years old or above	Reference	-	-	-
**BMI (kg/m^2)**				
18.5 to <25 (Healthy)	-0.05 (-0.11 to 0.01)	0.041	-1.20(10.00)	0.258
25 to <30 (Overweight)	-0.06 (-0.14 to 0.02)	0.050	-1.22(10.00)	0.249
30 or higher (Obese)	Reference	-	-	-
**Has chronic disease(s)**				
No	-0.04 (-0.13 to 0.06)	0.061	-0.62(10.00)	0.547
Yes	Reference	-	-	-
**Sleep apnea severity (AHI)**				
Mild or moderate	0.03 (-0.02 to 0.09)	0.036	0.89(10.00)	0.393
Severe	Reference	-	-	-
**Average nocturnal sleep time**				
<7 hours	Reference	-	-	-
7 hours or above	-0.01 (-0.08 to 0.06)	0.045	-0.27(10.00)	0.794
**CPAP compliance**				
Poor (<70%)	Reference	-	-	-
Good (70% and above)	-0.01 (-0.07 to 0.06)	0.043	-0.15(10.00)	0.884
**Condition**				
Pre-CPAP treatment	Reference	-	-	-
Post-CPAP treatment	0.00 (-0.01 to 0.01)	0.006	0.50(89.00)	0.616

Random effects (intercept): subject, SD = 0.064; residual, SD = 0.029

Supplementary Table S3. LMM between PVT RRT and age, BMI, chronic disease status, sleep apnea severity, average sleep time, CPAP compliance, and condition
Fixed effects

	**Estimate (95% CI)**	**SE**	***t*(df)**	***P***
**Intercept**	3.01 (2.69 to 3.34)	0.210	14.34(10.13)	**<0.001**
**Age at enrolment**				
<63 years old	0.34 (-0.02 to 0.69)	0.230	1.47(10.00)	0.171
63 years old and above	Reference	-	-	-
**BMI (kg/m^2)**				
18.5 to <25 (Healthy)	0.29 (-0.11 to 0.69)	0.260	1.13(10.00)	0.287
25 to <30 (Overweight)	0.41 (-0.08 to 0.90)	0.318	1.30(10.00)	0.224
30 or higher (Obese)	Reference	-	-	-
**Has chronic disease(s)**				
No	0.30 (-0.31 to 0.90)	0.392	0.76(10.00)	0.466
Yes	Reference	-	-	-
**Sleep apnea severity (AHI)**				
Mild or moderate	-0.18 (-0.54 to 0.18)	0.234	-0.76(10.00)	0.462
Severe	Reference	-	-	-
**Average nocturnal sleep time**				
<7 hours	Reference	-	-	-
7 hours and above	0.04 (-0.41 to 0.49)	0.290	0.13(10.00)	0.899
**CPAP compliance**				
Poor (<70%)	Reference	-	-	-
Good (70% and above)	0.01 (-0.42 to 0.44)	0.277	0.03(10.00)	0.973
**Condition**				
Pre-CPAP treatment	Reference	-	-	-
Post-CPAP treatment	-0.01 (-0.07 to 0.06)	0.033	-0.19(89.00)	0.847

Random effects (intercept): subject, SD = 0.408; residual, SD = 0.174

Supplementary Table S4. LMM between PVT lapses and age, BMI, chronic disease status, sleep apnea severity, average sleep time, CPAP compliance, and condition
Fixed effects

	Estimate (95% CI)	SE	t(df)	P
**Intercept**	7.97 (1.92 to 14.01)	3.910	2.04(10.28)	0.068
**Age at enrolment**				
<63 years old	-3.18 (-9.75 to 3.39)	4.258	-0.75(10.00)	0.473
63 years old or above	Reference	-	-	-
**BMI (kg/m^2)**				
18.5 to <25 (Healthy)	-6.09 (-13.54 to 1.36)	4.827	-1.26(10.00)	0.236
25 to <30 (Overweight)	-3.64 (-12.73 to 5.46)	5.895	-0.62(10.00)	0.551
30 or higher (Obese)	Reference	-	-	-
**Has chronic disease(s)**				
No	-2.21 (-13.41 to 9.00)	7.263	-0.30(10.00)	0.768
Yes	Reference	-	-	-
**Sleep apnea severity (AHI)**				
Mild or moderate	5.60 (-1.08 to 12.27)	4.328	1.29(10.00)	0.225
Severe	Reference	-	-	-
**Average nocturnal sleep time**				
<7 hours	Reference	-	-	-
7 hours or above	-2.76 (-11.05 to 5.54)	5.377	-0.51(10.00)	0.619
**CPAP compliance**				
Poor (<70%)	Reference	-	-	-
Good (70% and above)	-1.64 (-9.56 to 6.27)	5.129	-0.32(10.00)	0.755
**Condition**				
Pre-CPAP treatment	Reference	-	-	-
Post-CPAP treatment	-0.50 (-2.31 to 1.31)	0.917	-0.55(89.00)	0.587

Random effects (intercept): subject, SD = 7.421; residual, SD = 4.764
